# Supplementary material for: Large vision model framework for automated C. elegans analysis: From static morphometry to dynamic neural activity
Source: PLoS Comput Biol. 2026 Jul 22;22(7):e1013441. doi: 10.1371/journal.pcbi.1013441 (PMC13492816; doi:10.1371/journal.pcbi.1013441)
Supplement: Supplementary file 1 [file pcbi.1013441.s001.pdf]

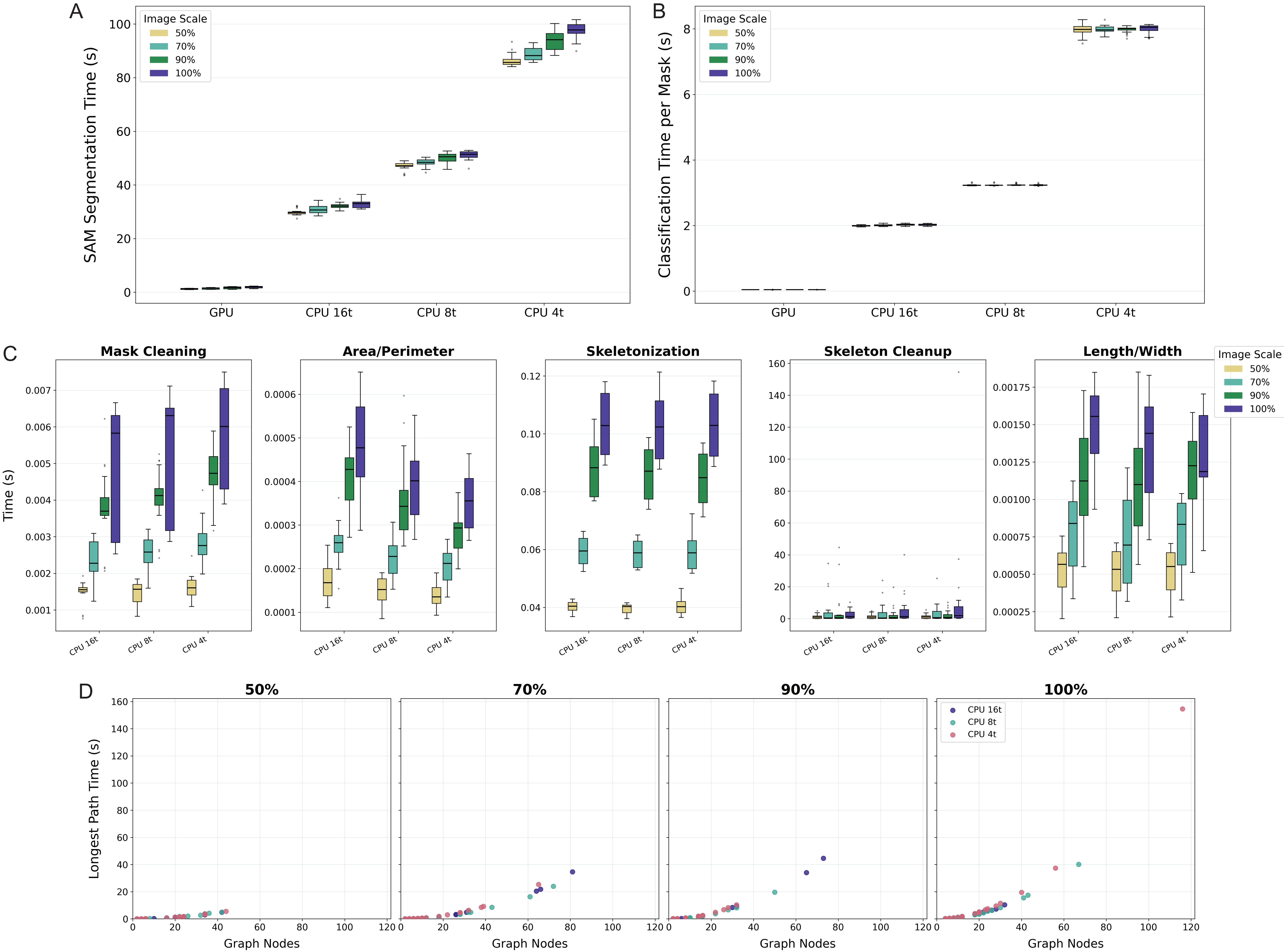

A
SAM Segmentation Time (s)
Image Scale
50%
70%
90%
100%
GPU
CPU 16t
CPU 8t
CPU 4t
B
Classification Time per Mask (s)
C
Mask Cleaning
Area/Perimeter
Skeletonization
Skeleton Cleanup
Length/Width
Time (s)
D
50%
70%
90%
100%
Longest Path Time (s)
Graph Nodes
