# Supplementary material for: Large vision model framework for automated C. elegans analysis: From static morphometry to dynamic neural activity
Source: PLoS Comput Biol. 2026 Jul 22;22(7):e1013441. doi: 10.1371/journal.pcbi.1013441 (PMC13492816; doi:10.1371/journal.pcbi.1013441)
Supplement: Supplementary file 2 [file pcbi.1013441.s002.pdf]

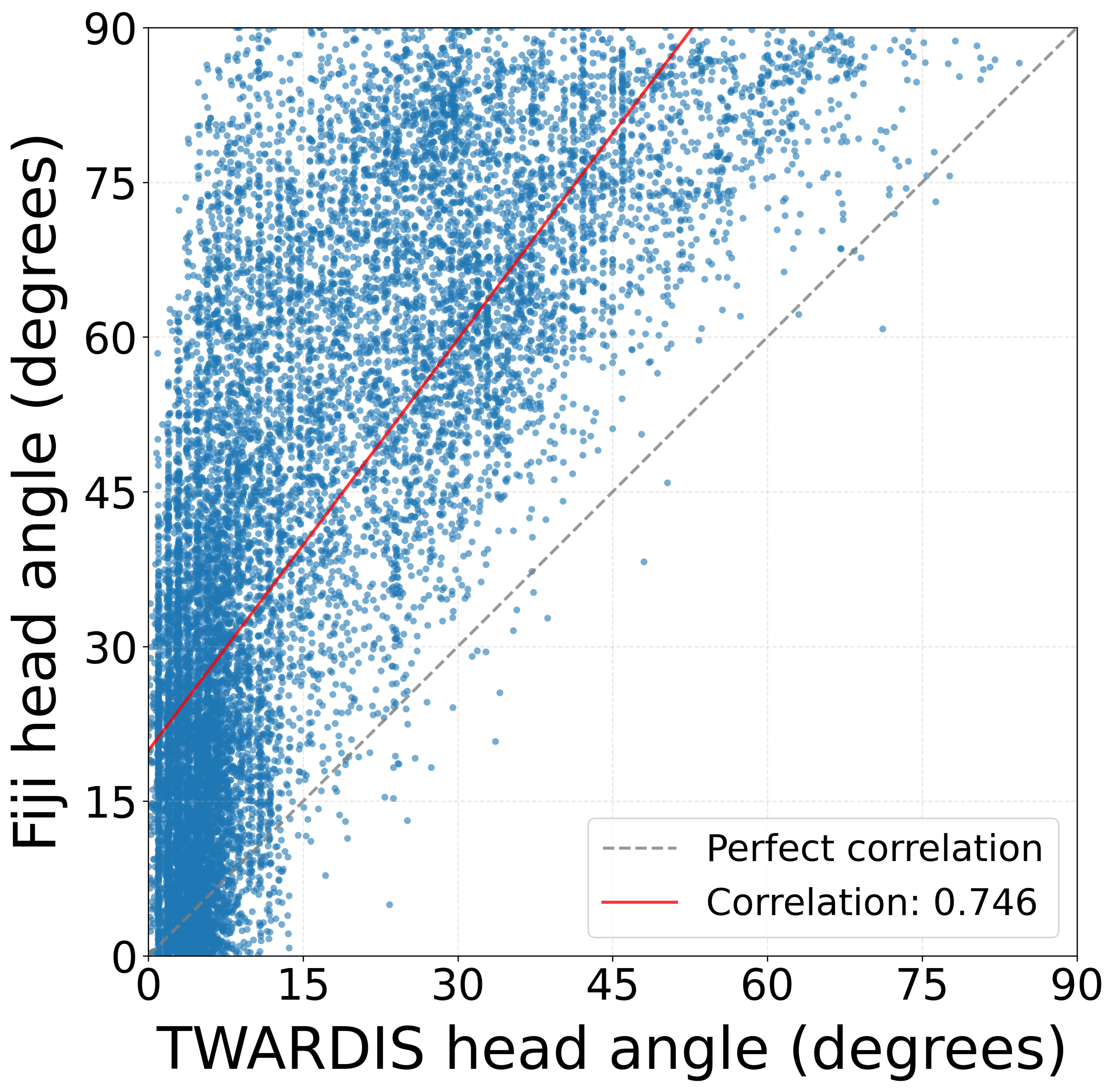
90
75
60
45
30
15
0
Fiji head angle (degrees)
0
15
30
45
60
75
90
TWARDIS head angle (degrees)
Perfect correlation
Correlation: 0.746
