# Supplementary material for: Large vision model framework for automated C. elegans analysis: From static morphometry to dynamic neural activity
Source: PLoS Comput Biol. 2026 Jul 22;22(7):e1013441. doi: 10.1371/journal.pcbi.1013441 (PMC13492816; doi:10.1371/journal.pcbi.1013441)
Supplement: Supplementary file 3 [file pcbi.1013441.s003.pdf]

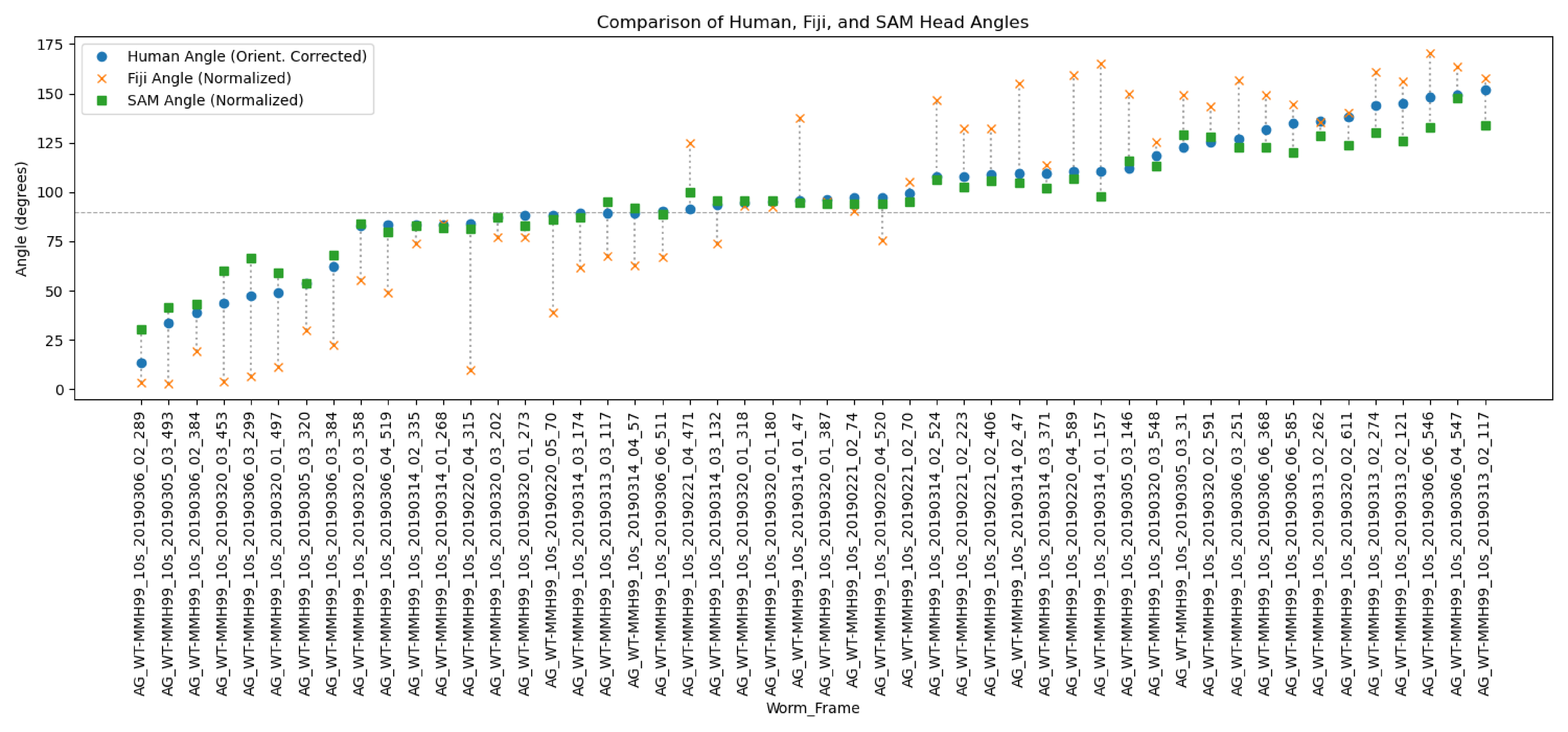

Comparison of Human, Fiji, and SAM Head Angles
Human Angle (Orient. Corrected)
Fiji Angle (Normalized)
SAM Angle (Normalized)
Angle (degrees)
Worm_Frame
AG_WT-MMH99_10s_20190306_02_289
AG_WT-MMH99_10s_20190305_03_493
AG_WT-MMH99_10s_20190306_02_384
AG_WT-MMH99_10s_20190320_03_453
AG_WT-MMH99_10s_20190306_03_299
AG_WT-MMH99_10s_20190320_01_497
AG_WT-MMH99_10s_20190305_03_320
AG_WT-MMH99_10s_20190306_03_384
AG_WT-MMH99_10s_20190320_03_358
AG_WT-MMH99_10s_20190306_04_519
AG_WT-MMH99_10s_20190314_02_335
AG_WT-MMH99_10s_20190314_01_268
AG_WT-MMH99_10s_20190220_04_315
AG_WT-MMH99_10s_20190320_03_202
AG_WT-MMH99_10s_20190320_01_273
AG_WT-MMH99_10s_20190220_05_70
AG_WT-MMH99_10s_20190314_03_174
AG_WT-MMH99_10s_20190313_03_117
AG_WT-MMH99_10s_20190314_04_57
AG_WT-MMH99_10s_20190306_06_511
AG_WT-MMH99_10s_20190221_04_471
AG_WT-MMH99_10s_20190314_03_132
AG_WT-MMH99_10s_20190320_01_318
AG_WT-MMH99_10s_20190320_01_180
AG_WT-MMH99_10s_20190314_01_47
AG_WT-MMH99_10s_20190320_01_387
AG_WT-MMH99_10s_20190221_02_74
AG_WT-MMH99_10s_20190220_04_520
AG_WT-MMH99_10s_20190221_02_70
AG_WT-MMH99_10s_20190314_02_524
AG_WT-MMH99_10s_20190221_02_223
AG_WT-MMH99_10s_20190221_02_406
AG_WT-MMH99_10s_20190314_02_47
AG_WT-MMH99_10s_20190314_03_371
AG_WT-MMH99_10s_20190220_04_589
AG_WT-MMH99_10s_20190314_01_157
AG_WT-MMH99_10s_20190305_03_146
AG_WT-MMH99_10s_20190320_03_548
AG_WT-MMH99_10s_20190305_03_31
AG_WT-MMH99_10s_20190320_02_591
AG_WT-MMH99_10s_20190306_03_251
AG_WT-MMH99_10s_20190306_06_368
AG_WT-MMH99_10s_20190306_06_585
AG_WT-MMH99_10s_20190313_02_262
AG_WT-MMH99_10s_20190320_02_611
AG_WT-MMH99_10s_20190313_02_274
AG_WT-MMH99_10s_20190313_02_121
AG_WT-MMH99_10s_20190306_06_546
AG_WT-MMH99_10s_20190306_04_547
AG_WT-MMH99_10s_20190313_02_117
