# Supplementary material for: Large vision model framework for automated C. elegans analysis: From static morphometry to dynamic neural activity
Source: PLoS Comput Biol. 2026 Jul 22;22(7):e1013441. doi: 10.1371/journal.pcbi.1013441 (PMC13492816; doi:10.1371/journal.pcbi.1013441)
Supplement: Supplementary file 4 [file pcbi.1013441.s018.docx]

Guisnet and Hendricks’ “Large vision model framework for automated C. elegans analysis: From static morphometry to dynamic neural activity” presents ‘TWARDIS’, a python-based framework that makes timely use of large vision models (SAM and SAM2) to develop a fine-tuned vision transformer classifier which automates the segmentation steps of *C. elegans* tracking. They do a good job of demonstrating the utility of TWARDIS across increasingly complex applications:

1. Static overlapping worms – segmentation is near-perfect (0.99 correlation)
2. Swimming – high-fidelity posture extraction when worms only occupy a small area (~0.25%) of the field of view (FOV)
3. Crawling – robust posture and trajectory tracking can be derived from low-resolution, wide-field videos captured using inexpensive hardware
4. Calcium imaging – achieving precise segmentation of neural compartments in semi-restricted worms while avoiding ROI-induced signal flattening and deriving more biologically accurate head angle measurements

The described approach helps to overcome the fragility of some traditional threshold-based segmentation techniques. Helping to bridge the trade-off between throughput, precision and accessibility whilst working to develop a future-proof toolbox of segmentation techniques that can be universally applied to a wide-range of applications. Ultimately this modular framework will help to reduce some of the bottlenecks in worm phenotyping, decrease researcher bias and imoprove reproducibility across studies.

Although a seemingly powerful approach, I believe that this impact of the studies’ findings would be significantly bolstered if the authors could address the following:

- The methods are primarily demonstrated on wild-type C. elegans within a limited set of recording setups, and while TWARDIS does support diverse applications (the four points listed above), its scope excludes important experimental contexts like multi-worm tracking (see below), population-level analyses, or free-moving calcium imaging. The manuscripts primary claim is that TWARDS has broad applicability. Therefore, the manuscript would be strengthened by including (or at least discussing) validation on mutant strains or non-N2 wild-type isolates, additional multi-worm scenarios, and datasets generated by external labs (e.g., from open repositories). Such additions, together with a more concrete roadmap for modular extensions, would better demonstrate the generalisability and robustness of TWARDIS across segmentation tasks and experimental contexts.
- Further to the point above, one of the key areas I would like to see addressed (or at the very least discussed) is tracking multiple crawling worms. This is an extremely active field of research and key conclusions is that TWARDIS can be used to address the problem of overlapping/colliding worms for static images. The paper would certainly benefit by referencing to the ‘DeepTagleCrawl’ model (PMCID: PMC12360645) that has recently been shown to reduce tracking failure rates and produce gap-free worm trajectories during object collision or self-intersecting postures.
- The system leverages SAM/SAM2, which can be computationally demanding. Although CPU compatibility is claimed, runtime performance benchmarks (CPU vs GPU, dataset size scaling) are not provided. Including this information would allow readers to better judge the accessibility of TWARDIS for labs with modest resources.
- Similar to above, there is mention that the automation of segmentation makes processing time irrelevant. While I see the point being made, a direct comparison of mask generation time between SAM and other commonly used thresholding methods would be beneficial. Some researchers rely on the real-time processing of datasets as they are generated to keep pace with acquisition of wet-lab experiments. Informing readers whether the speed of TWARDIS/vision transformer-based models can, or cannot, keep pace with existing methods is crucial to enhance uptake in the field.
- The fine-tuned worm classifier shows ~5% false negatives on a second dataset, suggesting some overfitting. A discussion on strategies for expanding the training dataset (e.g., use of publicly available annotated datasets, synthetic data augmentation) would strengthen confidence in cross-setup applicability.
- The correlation values (e.g., 0.99 with manual segmentation) are impressive, but broader benchmarking against existing state-of-the-art worm analysis tools (e.g., Celeganser, WorMachine, CeleST, Tierpsy) would help contextualize TWARDIS’ performance and highlight where it provides the most substantial gains over other existing approaches.
- In all figures where TWARDIS is compared to other thresholding methods, please clarify what method is being used. Please also update the methods section to include how this/these comparison methods were performed.
- The approach towards analysing structures that move and have inconsistent geometries is effective and powerful for the biologically relevant interpretation of more complex tracking scenarios. Has this approach been tested on a different fluorescently labelled dataset? It would be a brilliant proof-of-concept to see if the same performance is achieved when applying TWARDIS to a similar challenge. Perhaps even reanalysing a similar dataset generated by a different group?
- This improved method of calculating head bends seems more effective than using an in-built ImageJ classifier. Can you also state what classifier was used for this? Also, please include a comparison between manually measured head angles the two automated methods.
- In general, TWARDIS presents a step-forward in developing the next stage of generalised and robust worm tracking solutions. I like the forward-thinking approach to ensure that this tool will remain future proof, and the plan to develop and distribute a more user-friendly version of TWARDIS. However, I think that some comment towards the limitations of this tool compared to existing methods should also be discussed in the conclusions.

Minor Comments:

- It is discussed that SAM can be used to identify cutouts of worms across all stages of life. In Fig 1D and 1E only the larger adult worms are identified in the TWARDIS overlay images. This figure legend does mention that it shows ‘age-based’ TWARDIS extraction. Please clarify what this is to avoid any confusion and explain to readers why the younger worms are not segmented.
- Further to above, can the methods section be updated to explain this ‘age-based’ classifier? If it uses morphological analysis, some discussion of how this may be affected when tracking mutant worms with morphological differences may be warranted.
- The mention of parallelization is nice. However, it is worth mentioning that many other worm tracking methods e.g. Multi-worm tracker and WormYOLO, are also parallelizable.
- In the legends for Figures 2, 3 and 4, it is state that SAM is used to track the worm objects. Should this not be ‘SAM2’ as they are videos?
- The ability to derive a broad feature set from a minimal hardware set up is a strong benefit for using TWARDIS. A readily searchable list/table of feature outputs from the segmentation and tracking toolbox would be beneficial for those wanting to try it for themselves. This could be included in the methods section, or as a ‘ReadMe’ file in the GitHub repository.
- While the GitHub repository is linked, information on documentation, tutorials, or example pipelines is minimal in the paper. Pointing readers to usage guides would enhance reproducibility. I do understand that this may be beyond the scope of the manuscript in its current form but just wanted to make the authors aware that it should be delivered alongside the full Python package.
- Terms like “prompt frame” or “prompt pool” for guiding SAM segmentation may be unclear to readers less familiar with foundation models- perhaps these could be defined in a short glossary of terms. Furthermore, clarification of what the ‘generic prompt frame’ used for the 4 different tracking scenarios/an example of these in the supplementary material section would improve accessibility and reproducibility.
- Comparisons between TWARDIS and thresholding are shown, but quantitative metrics (e.g., Intersection of Union, Dice scores) are not consistently provided alongside visual examples. Including these would make the claims more rigorous.
- The reliance on specific PyTorch versions, SAM2 checkpoints, and environment management tools could be barriers for less computationally experienced labs. The authors could note whether Docker/Conda environments are planned for future versions.
- The “Author Summary” is well written but could briefly mention computational requirements, as accessibility is a central theme to the manuscript.
