# Supplementary material for: Large vision model framework for automated C. elegans analysis: From static morphometry to dynamic neural activity
Source: PLoS Comput Biol. 2026 Jul 22;22(7):e1013441. doi: 10.1371/journal.pcbi.1013441 (PMC13492816; doi:10.1371/journal.pcbi.1013441)
Supplement: Supplementary file 5 [file pcbi.1013441.s020.docx]

**Response to Reviews**
We are grateful to the reviewers for the constructive and detailed feedback, which has substantially improved the clarity and rigor of our work. We have carefully addressed all comments, and a point-by-point response is available below.

**Reviewer #1**

This manuscript presents TWARDIS, an automated analysis framework based on large vision models (SAM, SAM2, and a Vision Transformer classifier) for the morphological, behavioral, and neural activity analysis of C. elegans. The novelty of this work lies in integrating general-purpose computer vision models into worm research, addressing limitations of traditional methods under noisy, overlapping, low-resolution, and complex dynamic conditions. The manuscript is well structured, the experimental design is clear, and the results convincingly demonstrate the method’s effectiveness.

Overall, the paper exhibits strong technical innovation and clear potential for practical applications, making it valuable to both the computational biology and C. elegans research communities. However, improvements are required in terms of generalization validation, comparisons with existing tools, multi-worm scenarios, and multi-metric quantitative evaluation. I therefore recommend major revision before acceptance.

Specific comments and suggestions

**R1.1**. Generalization and failure cases

In static multi-worm morphology analysis, the authors tested a second imaging setup, but the overall validation sample size remains limited. It is recommended to test on public datasets or larger multi-source data (e.g., WormAtlas, Multi-Worm-Lifespan) to demonstrate generalization. Additionally, how does the system perform under severe overlaps, blur, or occlusions? Report standard segmentation metrics and distributional result rather than single R-value.

***Sample sizes.*** *We believe there may be a misunderstanding regarding sample sizes. The n = 21 reported in Fig 1G corresponds specifically to the manual segmentation comparison, where an expert manually traced worm outlines to serve as quantitative comparison. The actual datasets analyzed by TWARDIS are substantially larger: the first dataset comprised 237 images and the second 343 images, with images containing between 1 and 5 worms each. In total, TWARDIS segmented and classified 675 worms across these two datasets. We also note that the two datasets were acquired on drastically different microscopes (Olympus MVX10 with CCD camera vs. Leica DMI6000B with spinning-disk confocal and EM-CCD with image tiling), with different sample preparation methods (sodium azide immobilization on agarose pads vs. levamisole between slide and coverslip), different magnifications, and different software. We believe our two-setup validation, with its variation in optics, preparation, and acquisition software, provides a stringent and relevant test of generalization. We agree the sample sizes were missing from the manuscript, and we have made revisions to include a table of sample sizes per developmental stage for both datasets to make this information explicit.
→ Manuscript line: 147*

***Performance under challenging conditions.*** *Figs 1B–F directly illustrate TWARDIS performance under what we believe are the conditions the reviewer describes. These panels include examples of completely overlapping worms, worms touching eggs and debris, not perfectly focused elements, and noisy backgrounds. These are all cases where thresholding (third row) fails to produce a correct segmentation without manual input. If the reviewer is referring to extreme overlap/blur conditions, we would like to clarify that there is a fundamental physical limit to what any segmentation method can recover: if an object is so severely occluded or blurred that it is no longer visually distinguishable, no segmentation approach can faithfully delineate the missing elements. Attempting to “complete” heavily occluded worms (amodal completion) is a distinct problem beyond object segmentation that introduces significant risk in a scientific context, as inferred morphology could introduce systematic measurement errors, particularly for morphologically abnormal mutants. We add an explicit note on this as a potential avenue for future work, and cite recent relevant models as starting points.
→ Manuscript lines: 480-487*

***Segmentation metrics.*** *We appreciate this suggestion. We note that standard computer vision metrics such as IoU are typically used to evaluate segmentation models against manual pixel-level annotations considered “ground truths” (a good caution on the validity of those “ground truths” is available here* [*https://arxiv.org/abs/1808.03575*](https://arxiv.org/abs/1808.03575)*, particularly the Semi-Supervision section of 4.2, and a more recent investigation here* [*https://arxiv.org/pdf/2403.18819*](https://arxiv.org/pdf/2403.18819)*). However, as we illustrate in Fig 1H, there are many situations where there is no objective ground truth for where the edge of an object begins or ends. Edge placement depends on focus quality, local contrast, and the subjective judgment of the annotator. Metrics such as IoU implicitly assume the existence of a single correct segmentation, and pixel-level disagreement with the reference is treated as error. In practice, for images where the object/worm boundary is ambiguous over several pixels, multiple valid segmentations exist, and apparent "errors" may simply reflect equally defensible edge placements. TWARDIS is also not a segmentation model itself; it is a pipeline built upon the SAM segmentation models, whose pixel-level segmentation performance has been extensively benchmarked elsewhere (*[*https://arxiv.org/abs/2304.02643*](https://arxiv.org/abs/2304.02643) *and* [*https://arxiv.org/abs/2408.00714*](https://arxiv.org/abs/2408.00714)*). Our validation instead focuses on the biologically relevant question: does the pipeline produce measurements equivalent to what an expert would obtain if they could afford to manually segment every worm one by one? The R = 0.999 correlation with manual expert segmentation demonstrates that TWARDIS faithfully reproduces expert-level morphological feature extraction, which is ultimately what researchers need from such a tool. To provide more scale to the comparison metric, we have added the mean ± SD of per-worm percent area differences between TWARDIS and manual segmentation in the revised manuscript, but add caution to the interpretation as explained above. We can also increase the number of manually segmented images to count towards this metric and Figure 1G if the reviewers think this will strengthen the conclusions.
→ Manuscript lines: 566-568*

**R1.2**. Dynamic multi-worm analysis

While static segmentation supports several worms, the dynamic swimming/crawling analysis is restricted to single worms. It would strengthen the work to include experiments with multiple worms in motion and quantify the important results, such as frame-retention rates.

*We agree that shape-level multi-worm tracking is indeed an exciting direction with the potential to unlock rich behavioral datasets, particularly for social behavior, population-level phenotyping, or high-throughput screening. We share the reviewer's enthusiasm for extending TWARDIS in this direction. However, we respectfully argue that this extension is beyond the scope of the present work, as it represents a fundamentally different technical challenge that warrants its own dedicated study.*

*The central contribution of this manuscript is to demonstrate that foundation vision models can replace traditional thresholding-based segmentation across a range of C. elegans imaging modalities, eliminating many bottlenecks in researcher time, equipment requirements, and data quality. We demonstrate this across four modalities of increasing complexity.*

*Multi-worm tracking introduces qualitatively different challenges that go well beyond segmentation: robust identity maintenance across frames, resolution of physical overlaps between moving individuals, re-identification after occlusion events, scalable skeleton assignment… none of which are sufficiently addressed by solely improving the quality of the segmentation output. Doing this will require dedicated model architectures and tracking strategies (e.g.: memory-augmented multi-object trackers, amodal completion networks, specialized pose estimation frameworks, etc.), several of which we cite as promising future directions in our Discussion (“Future iterations could aim to incorporate multi-worm tracking, multi-well swimming, diverse neuronal recording conditions or even freely moving calcium activity as we presented in* [*[47]*](https://paperpile.com/c/Hhi4A9/BE2DI)*.” Lines 471-473, “Future dynamic multi-worm approaches should leverage recent computer vision approaches developed for similar problems, such as memory-augmented multi-object trackers, specialized pose estimation frameworks, and methods for skeleton extraction and multi-worm overlap resolution [48–51].” Lines 477-480). Incorporating these approaches to truly create an improved multi-worm tracker would constitute a substantial body of work on its own and would shift the focus of the paper away from its core contribution.*

*We also note that the C. elegans community employs a diversity of imaging and analysis paradigms (multi-well formats, freely moving calcium imaging, optogenetic stimulation assays, developmental time-lapse imaging, behavioral assays, and many others) each presenting their own technical demands. Multi-worm tracking is one of many possible compelling extensions.*

*We have expanded this section of the discussion for clarity.
→ Manuscript lines: 471-487*

**R1.3**. Comparisons with recent tools

The main results are compared by TWARDIS with thresholding. A more convincing evaluation would include comparisons with recent deep learning–based tools.

*We appreciate the opportunity to clarify our rationale for the comparison strategy. The central contribution of TWARDIS is at the segmentation step itself, which we identify as the foundational bottleneck in C. elegans image analysis pipelines. Our comparison against thresholding was therefore deliberate, as it represents the segmentation method used by virtually all existing tools in the field.*

*While several recent tools incorporate deep learning components, these components operate downstream of the initial segmentation rather than replacing it. Some examples:*

- *Banerjee, 2023: Segmentation with thresholding + fine-tuned YOLO for worm ID maintenance in multi-worm tracking.*
- *Barlow, 2022: Initial masks with thresholding + worm classifier for multi-well tracking.*
- *Alonso, 2023: Segmentation with thresholding + multi-worm overlap resolution with CNN.*
- *Weheliye, 2025: Fine-tune of Alonso, 2023.*
- *Hakim, 2018: Initial masks with thresholding + worm classifier.*
- *Hebert, 2021: Initial threshold based track with head/tail positions + CNN to resolve coils and self-overlaps.*

*Other approaches, which have replaced the segmentation step, used custom or fine-tuned models and reported subpar generalisation. Some examples:*

- *Bates, 2022: Fine-tuned FasterRCNN (5,000 fine-tuning images) (Fig 1B)*
- *Jung, 2021: Trained CNN classifier (16,000 training images) (Table 3)*

*In each of these cases, the segmentation itself (the step where TWARDIS makes its main contribution) is either performed by thresholding or requires extensive task-specific training data. Comparing TWARDIS segmentation against thresholding is therefore effectively comparing it against all tools of the first category as they have the same underlying segmentation approach. For the second category, the reliance on large condition-specific annotated datasets is precisely the generalization limitation we discuss in the Introduction (second to last paragraph): these approaches attempted to improve on the initial segmentation step, but reported limited performance when applied outside their training conditions, illustrating the challenges of this strategy. Comparing TWARDIS to such tools would be of limited comparative value, as they are themselves presented as requiring re-training across recording setups. The foundational SAM models’ pixel-level segmentation performance has been extensively benchmarked elsewhere (*[*https://arxiv.org/abs/2304.02643*](https://arxiv.org/abs/2304.02643) *and* [*https://arxiv.org/abs/2408.00714*](https://arxiv.org/abs/2408.00714)*), and we demonstrate its applicability and generalizability to worm objects in Figure 1. Our work shows that recent foundation vision models (we chose SAMs) already solve segmentation at a level that removes the requirement for task-specific training for these applications. A direct comparison to these deep learning tools would require either applying their pre-trained models to our data, which would confirm their stated generalization limitations, or retraining them on our data, which would address a fundamentally different question than the one posed by our work.*

*For other tools that apply deep learning to problems such as multi-worm identity maintenance across frames or overlap resolution, they address qualitatively different challenges than those tackled by the current TWARDIS pipelines, making direct benchmarking against them inappropriate.*

*To make this reasoning clearer to readers, we have added language to both the Introduction and Discussion explicitly noting this, and that this is why thresholding is the appropriate baseline for evaluating our approach.
→ Manuscript lines: 73-80, 477-480.*

**R1.4**. Calcium imaging evaluation metrics

In the calcium imaging section, performance is assessed only via Pearson correlation and MAD compared to Fiji manual ROI analysis. No metrics are provided for segmentation shape agreement or dynamics accuracy. Please consider including additional measures to more comprehensively validate the method.

*We appreciate this suggestion and the opportunity to clarify the logic of our validation approach for the calcium imaging analysis. We would first like to clarify that the Fiji-based extraction is not, and should not be, treated as a ground truth in our analysis: it is the established method whose limitations we are identifying. The correlation with the MAD and head angle comparisons (Fig 4E & 4I) are therefore not presented as accuracy metrics against a reference standard, but as quantitative demonstrations of where and why the two methods diverge. As we show in Figs 4D and 4F–H, the disagreements arise from known failure modes of the ROI-based approach (signal flattening from temporary ROI misalignment, particularly in the loop compartment, and head angle inflation from ellipse fitting). In this context, higher agreement with Fiji would indicate that TWARDIS is reproducing the same artifacts, which is not desired. Similar to our response to R1.1, comparing agreement between the two approaches would not be a measure of improvement, as the Fiji approach is not a true “ground truth”.*

*Regarding segmentation shape agreement specifically, similar to the static morphometry case discussed in R1.1, there is no “true” ground truth for frame-by-frame segmentation of dynamic sub-cellular compartments as the boundary is extremely blurry. The Fiji analysis is also carried out using a square or oval-shaped ROI, which does not correspond to the true shape of each subcellular compartment, making comparison again irrelevant. To that effect, compartment fluorescence is routinely extracted by selecting the top X% pixels, meaning ROI shape is already ignored.*

*That said, we have added supplemental analyses to the Results to more comprehensively characterize the divergence between the two methods. We now report the percent agreement (± SD) between Fiji and TWARDIS fluorescence intensity for each compartment (nrD, nrV, loop), and head angle. The low agreement for the head angle and loop is consistent with the signal flattening artifact and head angle inflation from ellipse fitting we identify in this section.
→ Manuscript lines: 351-362*

**R1.5**. Data sharing

To improve the impact of this study on the field, I highly recommend the authors to share the data later on.

*Our Dataset S4 (S4 Dataset. Fiji- and TWARDIS-extracted head bending and fluorescence time-series values of 10 frames-per-second one minute recordings of wild-type worms in a microfluidic device expressing GCaMP3.3 in RIA.) originally contained only the 4 recordings that were used in Figs 4C, 4D, 4F, 4G and 4H, but we have updated this dataset to contain all data (nrd, nrv, loop, head) for all 22 recordings used across Fig 4.
→ Manuscript lines: 994 - Supplemental dataset S4 updated.*

Minor comments

**R1.6.** Please improve quality of the figures. Almost all figures appear very blurry.

*We believe this is a result of submitting the figures as part of the PDF. We will gladly improve the quality of the final figures if requested by the Editor.
→ No change.*

**R1.7**. Please add a confusion matrix for shape classification in the method.

*Done!
→ Manuscript lines: 180 (Table 2).*

**R1.8**. Please put SAM/SAM2 hyperparameters and post-processing steps in a single table.

*We have added a new table that consolidates the SAM/SAM2 model variants, parameters, prompt strategies, and main post-processing steps across all four modalities. Because the post-processing pipelines differ across modalities to address modality-specific challenges, the table provides only the major steps with references to the relevant Methods subsections for full detail.
→ Manuscript lines: 180 (Table 2).*

**Reviewer #2**

TWARDIS (Tools for Worm Automated Recognition & Dynamic Imaging System) presents a potentially useful tool for automating the segmentation of dynamic structures in C. elegans imaging. The ability to perform rapid segmentation across different imaging modalities, including bright-field, dark-field, and wide-field fluorescence, is a valuable contribution.

Major Strengths and General Appreciation:

The development of robust segmentation tools is welcome, as it is often a time-consuming step in data processing. The application to RIA imaging is a compelling demonstration of the method's capabilities.

Major Concerns and Suggested Improvements:

My primary concerns center on the demonstrated scope of the method and the framing of its impact relative to the field's most persistent challenges.

**R2.1.** Robustness Under Suboptimal Conditions: While segmentation is critical, experienced practitioners often have reliable methods that work well under excellent imaging conditions. The true power of a new tool like TWARDIS would be most evident in challenging scenarios where image quality is poor. I strongly encourage the authors to demonstrate their method's performance and adaptability under a wider range of non-ideal conditions, such as varying illumination, signal-to-noise ratios, contrast, and more complicated backgrounds. This would significantly strengthen the claim of generalizability and be helpful for the community.

*We want to clarify that the data presented throughout the paper were not acquired under carefully optimized imaging conditions for this analysis: they are representative of routine laboratory practice with minimal preparation and were originally intended for manual analysis. Figs 1B–F include examples with overlapping worms, uneven illumination, out-of-focus elements, background debris, and variable noise levels, all of which cause thresholding-based methods to fail (third row). The swimming and crawling analyses (Figs 2 and 3) used inexpensive, minimal hardware (a single FLIR camera with standard LED lighting) where the worm occupied only ~0.25% of the field of view. The calcium imaging data (Fig 4) involved deforming neural structures in a moving animal. Across all modalities, TWARDIS produced accurate segmentations without frame rejection. Additionally, the two static imaging datasets were acquired on quite different systems: an Olympus compound microscope with a CCD camera versus a Leica inverted microscope with spinning-disk confocal and EM-CCD, and also used different sample preparations, magnifications, and acquisition software (revised Table 1).*

*We agree that experienced practitioners often have reliable methods to produce excellent imaging conditions and this is in fact a key motivation for this paper. Our tools are designed to substantially relax the requirements for imaging quality relative to thresholding-based approaches, reducing the time and cost spent on optimizing illumination, focus, and sample preparation. However, we do not claim that TWARDIS works (or aim to make it work) under arbitrarily degraded conditions. There is a physical limit below which an object is no longer visually distinguishable, and no segmentation method (including human) could objectively faithfully recover information that is not present in the image.*

*We also agree in principle that testing on external data sources is valuable. However, to our knowledge there are currently no standardized C. elegans imaging benchmarks with expert-validated ground-truth segmentations that would enable a rigorous quantitative comparison. Applying TWARDIS to images from other publications would provide a qualitative demonstration, but without reliable ground truth, we do not believe this would yield the kind of systematic evaluation the reviewer requests. A comprehensive meta-analysis across publicly available datasets, while an exciting prospect, would constitute a substantial effort beyond the scope of this paper. We believe the combination of our two-setup cross-validation, the manual segmentation comparison, and the new noise degradation analysis (below) provides a thorough characterization of TWARDIS performance and its limits for the current work. We note that a related point regarding sample sizes and generalization was raised by Reviewer 1 (see response R1.1), where we provide additional detail on dataset composition.*

*We have added a noise degradation analysis to make the degradation boundary more explicit. We applied progressive levels of Gaussian noise to our images and repeated the TWARDIS segmentation and area measurement pipeline, comparing results to the manual expert segmentation as in Fig 1G. This characterizes the boundary at which performance begins to degrade. This analysis has been incorporated into the revised Fig 1G with a new Table that breaks down the results, and is discussed in the Results section.
→ Manuscript lines: 168-179*

*We have also revised the manuscript to adjust the sections where misleading language incorrectly suggested universal robustness.*

*→ Manuscript lines: various qualifying language throughout*

**R2.2.** Addressing the True Bottlenecks in Behavioral Analysis: The abstract states that TWARDIS "removes critical bottlenecks...enabling researchers to focus on biological questions rather than technical image processing challenges." in C. elegans research. However, several more fundamental bottlenecks exist in quantitative behavioral analysis that are not primarily segmentation problems. These include:

Animal Occlusion: Overlapping worms or self-occlusion (e.g., head/tail overlapping with the body) frequently break automated tracking and centerline extraction pipelines.

Head/Tail Discrimination: Accurately assigning head and tail identity during complex maneuvers remains a challenge.

Trajectory Management: Collisions often force researchers to manually merge or discard worm trajectories.

Since these events typically require manual intervention, the claim of having "removed" the critical bottlenecks is an overstatement. I suggest the authors moderate this language or, better yet, demonstrate how TWARDIS's segmentation output might be uniquely robust in scenarios that lead to these failures.

*This comment was addressed in* ***R1.2****. In addition, we have adjusted phrases where misleading language incorrectly suggested all bottlenecks were solved.*

*→ Manuscript lines: various*

**R2.3.** Scope of Application in Neural Imaging: While the RIA imaging example is excellent, the challenges in calcium imaging of neurons in moving animals often extend far beyond segmentation. Critical issues like registering signals to a highly deformable tissue and extracting meaningful activity traces subject to significant motion artifact from intricate neurites require innovative solutions that are distinct from segmentation. Presenting RIA as a key example is effective, but framing it as a solution to the broader challenges in neural imaging may overpromise. Clarifying the specific problem TWARDIS solves within that pipeline would be helpful.

In summary, TWARDIS is a promising tool for segmentation. To maximize its impact, I believe the manuscript would benefit from a more truthful discussion of its capabilities in the context of the field's well-known, unsolved problems, and a demonstration of its robustness where existing methods typically fail.

*We agree with the reviewer that the challenges in neural calcium imaging extend well beyond segmentation, particularly in 3D imaging and freely moving animals. We did not intend to suggest that TWARDIS addresses all of these challenges. Rather, our calcium imaging application demonstrates that foundation model-based segmentation can solve a specific, but major, issue within the neural imaging pipeline: the accurate, frame-by-frame delineation of neural compartments without manual ROI adjustment. We have revised the text to more precisely frame our contribution as addressing the segmentation component of neural imaging analysis, and we expand the Discussion section to address this more explicitly as well.
→ Manuscript lines: e.g. 455-463*

**R2.4.** Code Accessibility and Documentation: To ensure the community can actually use and benefit from TWARDIS, the provided GitHub repository requires significant improvement. Currently, it lacks a clear README file with essential information such as:

Installation instructions (including a list of dependencies and their versions).

A basic "getting started" tutorial or example.

Documentation of the code's core functions and input/output formats.

Addressing this is crucial for the adoption and practical utility of the tool. A well-documented repository is as important as the algorithm itself for a tool intended to "remove bottlenecks."

*Done!
→* [*https://github.com/lillyguisnet/TWARDISv0.1/blob/main/README.md*](https://github.com/lillyguisnet/TWARDISv0.1/blob/main/README.md)

**Reviewer #3**

Guisnet and Hendricks’ “Large vision model framework for automated C. elegans analysis: From static morphometry to dynamic neural activity” presents ‘TWARDIS’, a python-based framework that makes timely use of large vision models (SAM and SAM2) to develop a fine-tuned vision transformer classifier which automates the segmentation steps of C. elegans tracking. They do a good job of demonstrating the utility of TWARDIS across increasingly complex applications:

1. Static overlapping worms – segmentation is near-perfect (0.99 correlation)

2. Swimming – high-fidelity posture extraction when worms only occupy a small area (~0.25%) of the field of view (FOV)

3. Crawling – robust posture and trajectory tracking can be derived from low-resolution, wide-field videos captured using inexpensive hardware

4. Calcium imaging – achieving precise segmentation of neural compartments in semi-restricted worms while avoiding ROI-induced signal flattening and deriving more biologically accurate head angle measurements

The described approach helps to overcome the fragility of some traditional threshold-based segmentation techniques. Helping to bridge the trade-off between throughput, precision and accessibility whilst working to develop a future-proof toolbox of segmentation techniques that can be universally applied to a wide-range of applications. Ultimately this modular framework will help to reduce some of the bottlenecks in worm phenotyping, decrease researcher bias and imoprove reproducibility across studies.

Although a seemingly powerful approach, I believe that this impact of the studies’ findings would be significantly bolstered if the authors could address the following:

**R3.1.** The methods are primarily demonstrated on wild-type C. elegans within a limited set of recording setups, and while TWARDIS does support diverse applications (the four points listed above), its scope excludes important experimental contexts like multi-worm tracking (see below), population-level analyses, or free-moving calcium imaging. The manuscripts primary claim is that TWARDS has broad applicability. Therefore, the manuscript would be strengthened by including (or at least discussing) validation on mutant strains or non-N2 wild-type isolates, additional multi-worm scenarios, and datasets generated by external labs (e.g., from open repositories). Such additions, together with a more concrete roadmap for modular extensions, would better demonstrate the generalisability and robustness of TWARDIS across segmentation tasks and experimental contexts.

*We believe the main concerns raised here have been substantively addressed in our responses to* ***R1.1****.,* ***R1.2****. and* ***R2.1.*** *We have still added some more explicit specifications in the Discussion.*
*→ Manuscript lines: 455-463*

**R3.2.** Further to the point above, one of the key areas I would like to see addressed (or at the very least discussed) is tracking multiple crawling worms. This is an extremely active field of research and key conclusions is that TWARDIS can be used to address the problem of overlapping/colliding worms for static images. The paper would certainly benefit by referencing to the ‘DeepTagleCrawl’ model (PMCID: PMC12360645) that has recently been shown to reduce tracking failure rates and produce gap-free worm trajectories during object collision or self-intersecting postures.

*We believe this comment has been addressed by our response to* ***R1.2****. Regarding, Weheliye et al. 2024, we agree that this contribution will likely be an essential part in building multi-worm analysis tools of great quality and it was already cited (reference #32).*
*→ No additional change.*

**R3.3.** The system leverages SAM/SAM2, which can be computationally demanding. Although CPU compatibility is claimed, runtime performance benchmarks (CPU vs GPU, dataset size scaling) are not provided. Including this information would allow readers to better judge the accessibility of TWARDIS for labs with modest resources.

Similar to above, there is mention that the automation of segmentation makes processing time irrelevant. While I see the point being made, a direct comparison of mask generation time between SAM and other commonly used thresholding methods would be beneficial. Some researchers rely on the real-time processing of datasets as they are generated to keep pace with acquisition of wet-lab experiments. Informing readers whether the speed of TWARDIS/vision transformer-based models can, or cannot, keep pace with existing methods is crucial to enhance uptake in the field.

*Done! We have also added model sizes for reference.*
*→ Manuscript lines: 572*

**R3.4.** The fine-tuned worm classifier shows ~5% false negatives on a second dataset, suggesting some overfitting. A discussion on strategies for expanding the training dataset (e.g., use of publicly available annotated datasets, synthetic data augmentation) would strengthen confidence in cross-setup applicability.

*Agreed! This was already discussed in the future directions section of the Discussion (lines 464-) and expanded in our response to* ***R2.1.***
*→ No additional change.*

**R3.5.** The correlation values (e.g., 0.99 with manual segmentation) are impressive, but broader benchmarking against existing state-of-the-art worm analysis tools (e.g., Celeganser, WorMachine, CeleST, Tierpsy) would help contextualize TWARDIS’ performance and highlight where it provides the most substantial gains over other existing approaches.

*This overlaps with comment* ***R1.3****.*
*→ No additional change.*

**R3.6.** In all figures where TWARDIS is compared to other thresholding methods, please clarify what method is being used. Please also update the methods section to include how this/these comparison methods were performed.

*This overlaps with comment* ***R1.3****. Methods were updated.*
*→ Manuscript lines: 518*

**R3.7.** The approach towards analysing structures that move and have inconsistent geometries is effective and powerful for the biologically relevant interpretation of more complex tracking scenarios. Has this approach been tested on a different fluorescently labelled dataset? It would be a brilliant proof-of-concept to see if the same performance is achieved when applying TWARDIS to a similar challenge. Perhaps even reanalysing a similar dataset generated by a different group?

*This comment overlaps with* ***R1.1****. and* ***R2.1.***	*→ No additional change.*

**R3.8.** This improved method of calculating head bends seems more effective than using an in-built ImageJ classifier. Can you also state what classifier was used for this? Also, please include a comparison between manually measured head angles the two automated methods.

*We believe there may be a misunderstanding that we would like to clarify. No classifier is used for head angle measurement in either method. The reviewer may be referring to our fine-tuned ViT classifier, which is used exclusively in the static morphometry pipeline for segment classification (Fig 1A), and is not involved in the calcium imaging analysis. In the RIA analysis, each video has similarities (a worm semi-restricted in a chip with the same fluorescent neuron). We can leverage this similarity and use generic prompt frames to guide the SAM model to segment our objects of interest (RIA and worm body, in this case). However, in the static morphometry context, each image is unique and varies greatly (worm size, position, number, image brightness, debris, size, etc.). Thus, there is no “similarity” that can be leveraged in this situation to provide the model with generic guiding prompt frames (examples of what we want to identify in the image). Thus, we ask the SAM model to find all objects in the image and then use our classifier to keep worms only. The Fiji-based head angle extraction uses ellipse fitting on a binary-thresholded image of the worm's head, as described in Ouellette et al. (2018), no classifier is involved. Our TWARDIS approach first uses whole-body segmentation followed by skeletonization to find the absolute head angle relative to the body midline. We have adjusted some language to make this clearer.*
*→ Manuscript lines: 338-350*

*Regarding comparison to manual angles, we have included a comparison to Fiji and TWARDIS methods against human-measured head angles for 50 randomly selected frames and updated the text. TWARDIS-extracted angles showed substantially closer agreement with human measurements than Fiji-extracted angles, as expected. However, we would like to emphasize that, similar to our discussion on ground truths in* ***R1.4****. and morphological segmentation (Fig 1H), human head angle measurement is itself subject to observer bias and line-placement ambiguity, and should not be treated as an absolute ground truth. While theoretically close – human-measured angles are not absolute truths, and thus these results should be interpreted with caution. The closer agreement between TWARDIS and human measurements is nonetheless consistent with the artifacts we identified in the ellipse-fitting approach (Figs 4F–I).*
*→ Manuscript lines: 357-362*

**R3.9.** In general, TWARDIS presents a step-forward in developing the next stage of generalised and robust worm tracking solutions. I like the forward-thinking approach to ensure that this tool will remain future proof, and the plan to develop and distribute a more user-friendly version of TWARDIS. However, I think that some comment towards the limitations of this tool compared to existing methods should also be discussed in the conclusions.

*We believe this concern was addressed in* ***R1.3****.*
*→ No additional change.*

Minor Comments:

**R3.10.** It is discussed that SAM can be used to identify cutouts of worms across all stages of life. In Fig 1D and 1E only the larger adult worms are identified in the TWARDIS overlay images. This figure legend does mention that it shows ‘age-based’ TWARDIS extraction. Please clarify what this is to avoid any confusion and explain to readers why the younger worms are not segmented.

*Done!*
*→ Manuscript lines: 467-470*

**R3.11.** Further to above, can the methods section be updated to explain this ‘age-based’ classifier? If it uses morphological analysis, some discussion of how this may be affected when tracking mutant worms with morphological differences may be warranted.

*Done!*
*→ Manuscript lines: 553-559*

**R3.12.** The mention of parallelization is nice. However, it is worth mentioning that many other worm tracking methods e.g. Multi-worm tracker and WormYOLO, are also parallelizable.

*We have specified explicitly that parallelization and GPU-enhancement are not exclusive of TWARDIS.*
*→ Manuscript lines: 449-452*

**R3.13.** In the legends for Figures 2, 3 and 4, it is state that SAM is used to track the worm objects. Should this not be ‘SAM2’ as they are videos?

*We meant SAM”s” as generally any video-compatible SAM version (e.g. SAM 3.1 released on March 27th:* [*https://ai.meta.com/blog/segment-anything-model-3/*](https://ai.meta.com/blog/segment-anything-model-3/)*). However, we agree clarity of what we did is paramount here! We updated the figures themselves and their legends.*
*→ Manuscript lines: various*

**R3.14.** The ability to derive a broad feature set from a minimal hardware set up is a strong benefit for using TWARDIS. A readily searchable list/table of feature outputs from the segmentation and tracking toolbox would be beneficial for those wanting to try it for themselves. This could be included in the methods section, or as a ‘ReadMe’ file in the GitHub repository.

*This was addressed in* ***R2.4.***	*→ No additional change.*

**R3.15.** While the GitHub repository is linked, information on documentation, tutorials, or example pipelines is minimal in the paper. Pointing readers to usage guides would enhance reproducibility. I do understand that this may be beyond the scope of the manuscript in its current form but just wanted to make the authors aware that it should be delivered alongside the full Python package.

*We believe ReadMe updated through* ***R2.4.*** *addresses this*.
*→ No additional change.*

**R3.16.** Terms like “prompt frame” or “prompt pool” for guiding SAM segmentation may be unclear to readers less familiar with foundation models- perhaps these could be defined in a short glossary of terms. Furthermore, clarification of what the ‘generic prompt frame’ used for the 4 different tracking scenarios/an example of these in the supplementary material section would improve accessibility and reproducibility.

*Done! We have kept the definitions brief as machine learning basics are beyond the scope of the publication.*
*→ Manuscript lines: 214*

**R3.17.** Comparisons between TWARDIS and thresholding are shown, but quantitative metrics (e.g., Intersection of Union, Dice scores) are not consistently provided alongside visual examples. Including these would make the claims more rigorous.

*This comment overlaps with* ***R1.1****. and* ***R1.4****.*
*→ No additional change.*

**R3.18.** The reliance on specific PyTorch versions, SAM2 checkpoints, and environment management tools could be barriers for less computationally experienced labs. The authors could note whether Docker/Conda environments are planned for future versions.

*We agree that keeping accessibility in mind is important. While containerized environments like Docker have been a common solution in the field, we have found that they introduce their own substantial learning curve (installation, platform-specific troubleshooting, etc.) that can be just as challenging for non-technical users as a standard Python installation. We have received feedback that tools relying on Docker or complex compiled environments have faced adoption challenges for this reason.*

*Fortunately, the landscape for running models locally has changed considerably in the (very) recent years, with streamlined installation procedures and reduced dependency conflicts now standard in major frameworks. Also, modern Python environment managers such as uv (*[*https://github.com/astral-sh/uv*](https://github.com/astral-sh/uv)*) have also dramatically simplified dependency management, enabling reproducible environments with minimal user effort and without the overhead of containers. In our ideal accessibility approach, we first plan to release TWARDIS as a standard Python package with managed model dependencies that will simplify installation and handle checkpoint downloads automatically. Second, for users who may have access to capable hardware but lack command-line experience, we believe a simple web-based graphical interface that runs locally and leverages the user's own computational resources would be the best approach. Recent developments that now enable browser-based access to local GPUs (*[*https://www.w3.org/TR/webgpu/*](https://www.w3.org/TR/webgpu/)*) are particularly promising in this regard, as they would allow GPU-accelerated analysis without requiring any familiarity with Python environments or package management. We believe this combination of access will ultimately serve a broader range of labs than containerization. We have added a note to the Discussion.*
*→ Manuscript lines: 457-459*

**R3.19.** The “Author Summary” is well written but could briefly mention computational requirements, as accessibility is a central theme to the manuscript.

*Done!*
*→ Manuscript lines: 60-63*
