# Supplementary material for: Large vision model framework for automated C. elegans analysis: From static morphometry to dynamic neural activity
Source: PLoS Comput Biol. 2026 Jul 22;22(7):e1013441. doi: 10.1371/journal.pcbi.1013441 (PMC13492816; doi:10.1371/journal.pcbi.1013441)
Supplement: Supplementary file 6 [file pcbi.1013441.s021.docx]

Dear Editor,

Thank you for the opportunity to provide a second revision to our manuscript “Large vision model framework for automated *C. elegans* analysis: From static morphometry to dynamic neural activity” (PCOMPBIOL-D-25-01659).

We are grateful to you and the reviewers for the constructive and detailed feedback throughout this process. We have carefully addressed all remaining comments and a point-by-point response is available below.

We believe these revisions address the reviewer’s main concern about comparison and scope.

We thank you again for your consideration and look forward to your response.

With best regards,

Aurélie Guisnet and Michael Hendricks

**Reviewer #1**

The authors have provided a careful and detailed point-by-point response to the reviewers’ comments and have made several valuable revisions to the manuscript. In particular, the revised version includes clearer dataset sample-size statistics, a noise degradation analysis, additional quantitative comparisons in the calcium imaging section, expanded supporting datasets, and a summary table of SAM/SAM2 parameters and post-processing steps across modalities. These revisions improve the clarity and completeness of the manuscript.

**R1.1** However, based on a concern raised by both Reviewer #1 and Reviewer #3 in the first round, I still remain concerned about one central issue. I find the authors’ rationale partially reasonable: if the manuscript were framed strictly as a segmentation-focused study demonstrating improvement over thresholding-based pipelines, then thresholding would be an acceptable primary baseline. However, the revised manuscript still makes broader comparative statements about the limitations of existing tools and the general advancement offered by TWARDIS. Under that framing, direct comparison against at least one or two representative recent methods remains warranted. Alternatively, if the authors prefer not to add such experiments, the comparative claims should be substantially softened so that the paper is positioned more explicitly as a proof-of-concept framework rather than as a head-to-head state-of-the-art evaluation.

*We have modified the language across the manuscript, particularly in the introduction, where we have further described the limitations of known deep-learning tools, defined the gap more clearly and more thoroughly explained our comparison to thresholding. We have also compared our crawling analysis and the Tierpsy tracker on analysis completeness (% of frames with no extracted data), since the Tierpsy tracker is currently among the most used in the field.*

**R1.2** Have the authors made all data and (if applicable) computational code underlying the findings in their manuscript fully available?

The PLOS Data policy requires authors to make all data and code underlying the findings described in their manuscript fully available without restriction, with rare exception (please refer to the Data Availability Statement in the manuscript PDF file). The data and code should be provided as part of the manuscript or its supporting information, or deposited to a public repository. For example, in addition to summary statistics, the data points behind means, medians and variance measures should be available. If there are restrictions on publicly sharing data or code —e.g. participant privacy or use of data from a third party—those must be specified.

No: Partially. The revised manuscript has improved transparency by providing clearer dataset statistics, expanded supporting datasets, and a summary of SAM/SAM2 parameters and post-processing steps. However, I am not fully certain that all underlying data and computational code necessary to reproduce the findings have been made fully available. I would recommend that the authors explicitly state where the full datasets, code, model parameters, and post-processing scripts are deposited, and ensure that these resources are accessible before publication.

*We have added to the GitHub repository the classifier fine-tuning script, and added in supplementary the datasets for the Tierpsy vs TWARDIS crawling comparison. We believe all data presented in the paper has been shared, but will gladly address it if needed.*
