# Supplementary material for: Large vision model framework for automated C. elegans analysis: From static morphometry to dynamic neural activity
Source: PLoS Comput Biol. 2026 Jul 22;22(7):e1013441. doi: 10.1371/journal.pcbi.1013441 (PMC13492816; doi:10.1371/journal.pcbi.1013441)
Supplement: Peer review history

# Peer review: Decision Letter 0

**DOI:** 10.1371/journal.pcbi.1013441.r001

**Authors:** Stacey Finley, Jian Liu

2 Oct 2025

PCOMPBIOL-D-25-01659

Large vision model framework for automated *C. elegans*  analysis: From static morphometry to dynamic neural activity

PLOS Computational Biology

Dear Dr. Hendricks,

Thank you for submitting your manuscript to PLOS Computational Biology. After careful consideration, we feel that it has merit but does not fully meet PLOS Computational Biology's publication criteria as it currently stands. Therefore, we invite you to submit a revised version of the manuscript that addresses the points raised during the review process.

Please submit your revised manuscript within 60 days Dec 02 2025 11:59PM. If you will need more time than this to complete your revisions, please reply to this message or contact the journal office at ploscompbiol@plos.org. When you're ready to submit your revision, log on to https://www.editorialmanager.com/pcompbiol/ and select the 'Submissions Needing Revision' folder to locate your manuscript file.

Please include the following items when submitting your revised manuscript:

* A rebuttal letter that responds to each point raised by the editor and reviewer(s). You should upload this letter as a separate file labeled 'Response to Reviewers'. This file does not need to include responses to formatting updates and technical items listed in the 'Journal Requirements' section below.

* A marked-up copy of your manuscript that highlights changes made to the original version. You should upload this as a separate file labeled 'Revised Manuscript with Track Changes'.

* An unmarked version of your revised paper without tracked changes. You should upload this as a separate file labeled 'Manuscript'.

If you would like to make changes to your financial disclosure, competing interests statement, or data availability statement, please make these updates within the submission form at the time of resubmission. Guidelines for resubmitting your figure files are available below the reviewer comments at the end of this letter

We look forward to receiving your revised manuscript.

Kind regards,

Jian Liu

Academic Editor

PLOS Computational Biology

Stacey Finley

Section Editor

PLOS Computational Biology

**Journal Requirements:**

1) We ask that a manuscript source file is provided at Revision. Please upload your manuscript file as a .doc, .docx, .rtf or .tex. If you are providing a .tex file, please upload it under the item type u2018LaTeX Source Fileu2019 and leave your .pdf version as the item type u2018Manuscriptu2019.

2) Please amend your detailed Financial Disclosure statement. This is published with the article. It must therefore be completed in full sentences and contain the exact wording you wish to be published.

1) State the initials, alongside each funding source, of each author to receive each grant. For example: "This work was supported by the National Institutes of Health (####### to AM; ###### to CJ) and the National Science Foundation (###### to AM)."

3) Please ensure that the funders and grant numbers match between the Financial Disclosure field and the Funding Information tab in your submission form. Note that the funders must be provided in the same order in both places as well. Currently, this grant number "PJT-155980" is missing from the the Financial Disclosure field.

Note: If the reviewer comments include a recommendation to cite specific previously published works, please review and evaluate these publications to determine whether they are relevant and should be cited. There is no requirement to cite these works unless the editor has indicated otherwise.

**Reviewers' comments:**

Reviewer's Responses to Questions

**Comments to the Authors:**

**Please note that one review is uploaded as an attachment.**

Reviewer #1: This manuscript presents TWARDIS, an automated analysis framework based on large vision models (SAM, SAM2, and a Vision Transformer classifier) for the morphological, behavioral, and neural activity analysis of C. elegans. The novelty of this work lies in integrating general-purpose computer vision models into worm research, addressing limitations of traditional methods under noisy, overlapping, low-resolution, and complex dynamic conditions. The manuscript is well structured, the experimental design is clear, and the results convincingly demonstrate the method’s effectiveness.

Overall, the paper exhibits strong technical innovation and clear potential for practical applications, making it valuable to both the computational biology and C. elegans research communities. However, improvements are required in terms of generalization validation, comparisons with existing tools, multi-worm scenarios, and multi-metric quantitative evaluation. I therefore recommend major revision before acceptance.

Specific comments and suggestions

1. Generalization and failure cases

In static multi-worm morphology analysis, the authors tested a second imaging setup, but the overall validation sample size remains limited. It is recommended to test on public datasets or larger multi-source data (e.g., WormAtlas, Multi-Worm-Lifespan) to demonstrate generalization. Additionally, how does the system perform under severe overlaps, blur, or occlusions? Report standard segmentation metrics and distributional result rather than single R-value.

2. Dynamic multi-worm analysis

While static segmentation supports several worms, the dynamic swimming/crawling analysis is restricted to single worms. It would strengthen the work to include experiments with multiple worms in motion and quantify the important results, such as frame-retention rates.

3. Comparisons with recent tools

The main results are compared by TWARDIS with thresholding. A more convincing evaluation would include comparisons with recent deep learning–based tools.

4. Calcium imaging evaluation metrics

In the calcium imaging section, performance is assessed only via Pearson correlation and MAD compared to Fiji manual ROI analysis. No metrics are provided for segmentation shape agreement or dynamics accuracy. Please consider including additional measures to more comprehensively validate the method.

5. Data sharing

To improve the impact of this study on the field, I highly recommend the authors to share the data later on.

Minor comments

1. Please improve quality of the figures. Almost all figures appear very blurry.

2. Please add a confusion matrix for shape classification in the method.

3. Please put SAM/SAM2 hyperparameters and post-processing steps in a single table.

Reviewer #2: TWARDIS (Tools for Worm Automated Recognition & Dynamic Imaging System) presents a potentially useful tool for automating the segmentation of dynamic structures in C. elegans imaging. The ability to perform rapid segmentation across different imaging modalities, including bright-field, dark-field, and wide-field fluorescence, is a valuable contribution.

Major Strengths and General Appreciation:

The development of robust segmentation tools is welcome, as it is often a time-consuming step in data processing. The application to RIA imaging is a compelling demonstration of the method's capabilities.

Major Concerns and Suggested Improvements:

My primary concerns center on the demonstrated scope of the method and the framing of its impact relative to the field's most persistent challenges.

Robustness Under Suboptimal Conditions: While segmentation is critical, experienced practitioners often have reliable methods that work well under excellent imaging conditions. The true power of a new tool like TWARDIS would be most evident in challenging scenarios where image quality is poor. I strongly encourage the authors to demonstrate their method's performance and adaptability under a wider range of non-ideal conditions, such as varying illumination, signal-to-noise ratios, contrast, and more complicated backgrounds. This would significantly strengthen the claim of generalizability and be helpful for the community.

Addressing the True Bottlenecks in Behavioral Analysis: The abstract states that TWARDIS "removes critical bottlenecks...enabling researchers to focus on biological questions rather than technical image processing challenges." in C. elegans research. However, several more fundamental bottlenecks exist in quantitative behavioral analysis that are not primarily segmentation problems. These include:

Animal Occlusion: Overlapping worms or self-occlusion (e.g., head/tail overlapping with the body) frequently break automated tracking and centerline extraction pipelines.

Head/Tail Discrimination: Accurately assigning head and tail identity during complex maneuvers remains a challenge.

Trajectory Management: Collisions often force researchers to manually merge or discard worm trajectories.

Since these events typically require manual intervention, the claim of having "removed" the critical bottlenecks is an overstatement. I suggest the authors moderate this language or, better yet, demonstrate how TWARDIS's segmentation output might be uniquely robust in scenarios that lead to these failures.

Scope of Application in Neural Imaging: While the RIA imaging example is excellent, the challenges in calcium imaging of neurons in moving animals often extend far beyond segmentation. Critical issues like registering signals to a highly deformable tissue and extracting meaningful activity traces subject to significant motion artifact from intricate neurites require innovative solutions that are distinct from segmentation. Presenting RIA as a key example is effective, but framing it as a solution to the broader challenges in neural imaging may overpromise. Clarifying the specific problem TWARDIS solves within that pipeline would be helpful.

In summary, TWARDIS is a promising tool for segmentation. To maximize its impact, I believe the manuscript would benefit from a more truthful discussion of its capabilities in the context of the field's well-known, unsolved problems, and a demonstration of its robustness where existing methods typically fail.

Code Accessibility and Documentation: To ensure the community can actually use and benefit from TWARDIS, the provided GitHub repository requires significant improvement. Currently, it lacks a clear README file with essential information such as:

Installation instructions (including a list of dependencies and their versions).

A basic "getting started" tutorial or example.

Documentation of the code's core functions and input/output formats.

Addressing this is crucial for the adoption and practical utility of the tool. A well-documented repository is as important as the algorithm itself for a tool intended to "remove bottlenecks."

Reviewer #3: I have uploaded my author review as an attachment.

**********

**Have the authors made all data and (if applicable) computational code underlying the findings in their manuscript fully available?**

The PLOS Data policy requires authors to make all data and code underlying the findings described in their manuscript fully available without restriction, with rare exception (please refer to the Data Availability Statement in the manuscript PDF file). The data and code should be provided as part of the manuscript or its supporting information, or deposited to a public repository. For example, in addition to summary statistics, the data points behind means, medians and variance measures should be available. If there are restrictions on publicly sharing data or code —e.g. participant privacy or use of data from a third party—those must be specified.

Reviewer #1: **No:** Caclium image has not been released yet.

Reviewer #2: Yes

Reviewer #3: Yes

**********

PLOS authors have the option to publish the peer review history of their article (what does this mean?). If published, this will include your full peer review and any attached files.

If you choose “no”, your identity will remain anonymous but your review may still be made public.

**Do you want your identity to be public for this peer review?**  For information about this choice, including consent withdrawal, please see our Privacy Policy.

Reviewer #1: No

Reviewer #2: No

Reviewer #3: **Yes:** Thomas J. O'Brien

[NOTE: If reviewer comments were submitted as an attachment file, they will be attached to this email and accessible via the submission site. Please log into your account, locate the manuscript record, and check for the action link "View Attachments". If this link does not appear, there are no attachment files.]

**Figure resubmission:**

While revising your submission, we strongly recommend that you use PLOS’s NAAS tool (https://ngplosjournals.pagemajik.ai/artanalysis) to test your figure files. NAAS can convert your figure files to the TIFF file type and meet basic requirements (such as print size, resolution), or provide you with a report on issues that do not meet our requirements and that NAAS cannot fix.

After uploading your figures to PLOS’s NAAS tool - https://ngplosjournals.pagemajik.ai/artanalysis, NAAS will process the files provided and display the results in the "Uploaded Files" section of the page as the processing is complete. If the uploaded figures meet our requirements (or NAAS is able to fix the files to meet our requirements), the figure will be marked as "fixed" above. If NAAS is unable to fix the files, a red "failed" label will appear above. When NAAS has confirmed that the figure files meet our requirements, please download the file via the download option, and include these NAAS processed figure files when submitting your revised manuscript.

**Reproducibility:**

To enhance the reproducibility of your results, we recommend that authors of applicable studies deposit laboratory protocols in protocols.io, where a protocol can be assigned its own identifier (DOI) such that it can be cited independently in the future. Additionally, PLOS ONE offers an option to publish peer-reviewed clinical study protocols. Read more information on sharing protocols at https://plos.org/protocols?utm_medium=editorial-email&utm_source=authorletters&utm_campaign=protocols

Attachment Submitted filename: Author Comments.docx

# Author comment: Author response to Decision Letter 1

**DOI:** 10.1371/journal.pcbi.1013441.r002

8 Apr 2026

Attachment Submitted filename: Reponse to Reviewers.docx

# Peer review: Decision Letter 1

**DOI:** 10.1371/journal.pcbi.1013441.r003

**Authors:** Stacey Finley, Jian Liu

5 May 2026

PCOMPBIOL-D-25-01659R1

Large vision model framework for automated C. elegans analysis: From static morphometry to dynamic neural activity

PLOS Computational Biology

Dear Dr. Hendricks,

Thank you for submitting your manuscript to PLOS Computational Biology. After careful consideration, we feel that it has merit but does not fully meet PLOS Computational Biology's publication criteria as it currently stands. Therefore, we invite you to submit a revised version of the manuscript that addresses the points raised during the review process.

Please submit your revised manuscript by Jul 05 2026 11:59PM. If you will need more time than this to complete your revisions, please reply to this message or contact the journal office at ploscompbiol@plos.org. When you're ready to submit your revision, log on to https://www.editorialmanager.com/pcompbiol/ and select the 'Submissions Needing Revision' folder to locate your manuscript file.

Please include the following items when submitting your revised manuscript:

* A letter that responds to each point raised by the editor and reviewer(s). You should upload this letter as a separate file labeled 'Response to Reviewers'. This file does not need to include responses to formatting updates and technical items listed in the 'Journal Requirements' section below.

* A marked-up copy of your manuscript that highlights changes made to the original version. You should upload this as a separate file labeled 'Revised Manuscript with Track Changes'.

* An unmarked version of your revised paper without tracked changes. You should upload this as a separate file labeled 'Manuscript'.

If you would like to make changes to your financial disclosure, competing interests statement, or data availability statement, please make these updates within the submission form at the time of resubmission. Guidelines for resubmitting your figure files are available below the reviewer comments at the end of this letter.

We look forward to receiving your revised manuscript.

Kind regards,

Jian Liu

Associate Editor

PLOS Computational Biology

Stacey Finley

Section Editor

PLOS Computational Biology

**Additional Editor Comments:**

There are a few minor concerns that, if addressed, would help finalize the manuscript for publication. Please consider these suggestions and incorporate them as appropriate.

**********

Note: If the reviewer comments include a recommendation to cite specific previously published works, please review and evaluate these publications to determine whether they are relevant and should be cited. There is no requirement to cite these works unless the editor has indicated otherwise.

**Reviewers' comments:**

Reviewer's Responses to Questions

Reviewer #1: The authors have provided a careful and detailed point-by-point response to the reviewers’ comments and have made several valuable revisions to the manuscript. In particular, the revised version includes clearer dataset sample-size statistics, a noise degradation analysis, additional quantitative comparisons in the calcium imaging section, expanded supporting datasets, and a summary table of SAM/SAM2 parameters and post-processing steps across modalities. These revisions improve the clarity and completeness of the manuscript.

However, based on a concern raised by both Reviewer #1 and Reviewer #3 in the first round, I still remain concerned about one central issue. I find the authors’ rationale partially reasonable: if the manuscript were framed strictly as a segmentation-focused study demonstrating improvement over thresholding-based pipelines, then thresholding would be an acceptable primary baseline. However, the revised manuscript still makes broader comparative statements about the limitations of existing tools and the general advancement offered by TWARDIS. Under that framing, direct comparison against at least one or two representative recent methods remains warranted. Alternatively, if the authors prefer not to add such experiments, the comparative claims should be substantially softened so that the paper is positioned more explicitly as a proof-of-concept framework rather than as a head-to-head state-of-the-art evaluation.

Reviewer #2: The author has satisfactorily addressed my concerns.

**********

**Have the authors made all data and (if applicable) computational code underlying the findings in their manuscript fully available?**

The PLOS Data policy requires authors to make all data and code underlying the findings described in their manuscript fully available without restriction, with rare exception (please refer to the Data Availability Statement in the manuscript PDF file). The data and code should be provided as part of the manuscript or its supporting information, or deposited to a public repository. For example, in addition to summary statistics, the data points behind means, medians and variance measures should be available. If there are restrictions on publicly sharing data or code —e.g. participant privacy or use of data from a third party—those must be specified.

Reviewer #1: **No:** Partially. The revised manuscript has improved transparency by providing clearer dataset statistics, expanded supporting datasets, and a summary of SAM/SAM2 parameters and post-processing steps. However, I am not fully certain that all underlying data and computational code necessary to reproduce the findings have been made fully available. I would recommend that the authors explicitly state where the full datasets, code, model parameters, and post-processing scripts are deposited, and ensure that these resources are accessible before publication.

Reviewer #2: None

**********

PLOS authors have the option to publish the peer review history of their article (what does this mean?). If published, this will include your full peer review and any attached files.

If you choose “no”, your identity will remain anonymous but your review may still be made public.

**Do you want your identity to be public for this peer review?**  For information about this choice, including consent withdrawal, please see our Privacy Policy.

Reviewer #1: No

Reviewer #2: No

[NOTE: If reviewer comments were submitted as an attachment file, they will be attached to this email and accessible via the submission site. Please log into your account, locate the manuscript record, and check for the action link "View Attachments". If this link does not appear, there are no attachment files.]

**Figure resubmission:**

While revising your submission, we strongly recommend that you use PLOS’s NAAS tool (https://ngplosjournals.pagemajik.ai/artanalysis) to test your figure files. NAAS can convert your figure files to the TIFF file type and meet basic requirements (such as print size, resolution), or provide you with a report on issues that do not meet our requirements and that NAAS cannot fix.

After uploading your figures to PLOS’s NAAS tool - https://ngplosjournals.pagemajik.ai/artanalysis, NAAS will process the files provided and display the results in the "Uploaded Files" section of the page as the processing is complete. If the uploaded figures meet our requirements (or NAAS is able to fix the files to meet our requirements), the figure will be marked as "fixed" above. If NAAS is unable to fix the files, a red "failed" label will appear above. When NAAS has confirmed that the figure files meet our requirements, please download the file via the download option, and include these NAAS processed figure files when submitting your revised manuscript.

**Reproducibility:**

To enhance the reproducibility of your results, we recommend that authors of applicable studies deposit laboratory protocols in protocols.io, where a protocol can be assigned its own identifier (DOI) such that it can be cited independently in the future. Additionally, PLOS ONE offers an option to publish peer-reviewed clinical study protocols. Read more information on sharing protocols at https://plos.org/protocols?utm_medium=editorial-email&utm_source=authorletters&utm_campaign=protocols

# Author comment: Author response to Decision Letter 2

**DOI:** 10.1371/journal.pcbi.1013441.r004

22 Jun 2026

Attachment Submitted filename: Response to Reviewers.docx

# Editor report: Decision Letter 2

**DOI:** 10.1371/journal.pcbi.1013441.r005

**Authors:** Stacey Finley, Jian Liu

26 Jun 2026

Dear Dr. Hendricks,

We are pleased to inform you that your manuscript 'Large vision model framework for automated C. elegans analysis: From static morphometry to dynamic neural activity' has been provisionally accepted for publication in PLOS Computational Biology.

Before your manuscript can be formally accepted you will need to complete some formatting changes, which you will receive in a follow up email. A member of our team will be in touch with a set of requests.

Please note that your manuscript will not be scheduled for publication until you have made the required changes, so a swift response is appreciated.

IMPORTANT: The editorial review process is now complete. PLOS will only permit corrections to spelling, formatting or significant scientific errors from this point onwards. Requests for major changes, or any which affect the scientific understanding of your work, will cause delays to the publication date of your manuscript.

Should you, your institution's press office or the journal office choose to press release your paper, you will automatically be opted out of early publication. We ask that you notify us now if you or your institution is planning to press release the article. All press must be co-ordinated with PLOS.

Thank you again for supporting Open Access publishing; we are looking forward to publishing your work in PLOS Computational Biology.

Best regards,

Jian Liu

Academic Editor

PLOS Computational Biology

Stacey Finley

Section Editor

PLOS Computational Biology

***********************************************************

The revisions have satisfactorily addressed all concerns. The manuscript is now ready for acceptance.

# Editor report: Acceptance letter

**DOI:** 10.1371/journal.pcbi.1013441.r006

**Authors:** Stacey Finley, Jian Liu

PCOMPBIOL-D-25-01659R2

Large vision model framework for automated C. elegans analysis: From static morphometry to dynamic neural activity

Dear Dr Hendricks,

I am pleased to inform you that your manuscript has been formally accepted for publication in PLOS Computational Biology. Your manuscript is now with our production department and you will be notified of the publication date in due course.

The corresponding author will soon be receiving a typeset proof for review, to ensure errors have not been introduced during production. Please review the PDF proof of your manuscript carefully, as this is the last chance to correct any errors. Please note that major changes, or those which affect the scientific understanding of the work, will likely cause delays to the publication date of your manuscript.

Soon after your final files are uploaded, unless you have opted out, the early version of your manuscript will be published online. The date of the early version will be your article's publication date. The final article will be published to the same URL, and all versions of the paper will be accessible to readers.

For Research, Software, and Methods articles, you will receive an invoice from PLOS for your publication fee after your manuscript has reached the completed accept phase. If you receive an email requesting payment before acceptance or for any other service, this may be a phishing scheme. Learn how to identify phishing emails and protect your accounts at https://explore.plos.org/phishing.

Thank you again for supporting PLOS Computational Biology and open-access publishing. We are looking forward to publishing your work!

With kind regards,

Kannan R K Kuppusamy, B.TECH BIOTECHNOLOGY

PLOS Computational Biology | Carlyle House, Carlyle Road, Cambridge CB4 3DN | United Kingdom ploscompbiol@plos.org | Phone +44 (0) 1223-442824 | ploscompbiol.org | @PLOSCompBiol
